# Why do understorey *Licuala* palm fruits turn from red to white and then black when ripe?

**DOI:** 10.1002/ece3.70249

**Published:** 2024-09-13

**Authors:** Kenneth B. H. Er, Derrick H. D. Nguyen, Yi Shuen Yeoh, Max D. Y. Khoo, Ruisheng Choo, Li Si Tay, Sun Yi Soh, Zaki Jamil, Wee Foong Ang, Adrian H. B. Loo

**Affiliations:** ^1^ National Parks Board Singapore Singapore

**Keywords:** arecaceae, bulbul, dispersal syndrome, fruit, *Licuala*, reflectance

## Abstract

*Licuala ferruginea* Becc., a tropical forest understorey palm, is observed to have fruits that appear red in colour when unripe, turning pink, then white, purple and finally black in colour as they ripen. We monitored 13 fruiting palms in rainforest fragments and recorded the consumption of fruits by animals via camera traps. We also documented the fruiting phenology of two palms in the nursery. In the rainforest fragments, a Cream‐vented Bulbul (*Pycnonotus simplex*) was observed plucking a mature purple fruit from a *L. ferruginea* palm, before flying away with the fruit in its beak. This was the only bird that was observed feeding on the mature fruit. A range of mammals, dominated by edge species such as the Long‐tailed Macaque and Wild Boar, were observed to consume *L. ferruginea* fruits indiscriminately across all five colour stages, thereby limiting the dispersal of the fruits. Forest bulbul gape sizes also matched the fruit size, suggesting that forest bulbuls are the likely dispersers of the palm in the original forest where edge species are not in high densities. We further posit that the initial phase of red fruits, with high contrasting red reflectance against a green foliage background, might be a form of early advertisement to birds. The fruit then turns pink and white, which have high green reflectance and is less contrasting, thereby reducing the conspicuity of the fruit. This allows the fruit to ripen with high fructose and glucose content, and turn purple and black, which are known visual cues for birds. This study provides indicative support for the dispersal syndrome hypothesis and highlights the potential effects of forest fragmentation on plant–frugivore interactions.

## Graphical abstract

We record the changes in colour, sugar content, reflectance and predation of the fruits of a tropical understorey palm, *Licuala ferruginea*, as they ripen. Based on these signatures, we posit that the fruits are likely dispersed by birds such as forest bulbuls, and are predated upon but ineffectually dispersed by edge forest mammal species such as wild boar and long‐tailed macaques. This studdy provides indicative support for the dispersal syndrome hypothesis and highlights the potential effects of forest fragmentation on plant–frugivore interactions.

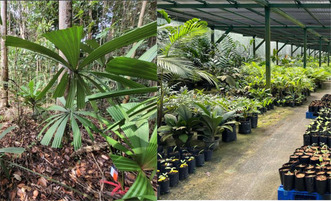

## 1 INTRODUCTION

Many plants are dependent on animals for dispersal, in a mutualistic interaction where animals are rewarded with nutrition as they disperse the seeds of plants. For this to happen, plants need to advertise their fleshy fruits to would‐be dispersers (Janson, 1983; Ridley, 1930). In recent years, studies have largely provided support for the dispersal syndrome hypothesis (Valenta et al., 2018; Valenta & Nevo, 2020).

Generally, bird dispersed fruits tend to be contrasting in colour (black, blue, red and white), whereas mammal dispersed fruits are more cryptic in colour (green, yellow, brown and orange) (Janson, 1983). Notwithstanding this, studies have found that selection of fruit colour alone is unlikely to drive the diversity and composition of the frugivore assemblage, which could be dependent more on fruit size (Brodie, 2017; Jordano, 1995). More recently, Sinnott‐Armstrong et al. (2021) showed that bird or mammal frugivory and abiotic factors are equally important interacting drivers of the fruit colour syndrome in 3163 fleshy‐fruited plant species globally. Specifically, they reported that contrastive bird‐associated fruit colours (red and black) increase with relative bird frugivore prevalence in warm areas, whereas these colours dominate even where mammal frugivores are more prevalent in cold places. This is consistent at least with experiments that showed bird preferences for reddish or black fruits in warm humid tropical areas (Duan et al., 2014).

This match between fruit colour and frugivore guilds has been further examined in terms of the colour vision capacities of the frugivores. Birds are tetrachromatic (able to detect colours like us, and wavelengths near the UV range), whereas most mammals are dichromatic (i.e. red‐green colour blind), although some primates are trichromatic and can see colours like us (Bennett & Théry, 2007; Jacobs, 2009). This has also re‐shaped the focus from fruit colour recognition per se to the ability to discern fruit colour contrast against the foliage background. Studies have shown that visually‐oriented birds are attracted more to red and black fruits as they give rise to high colour contrasts against the foliage in the background, compared with mammals which tend to be attracted to greener and less colour contrasting fruits (Melo et al., 2011; Nevo et al., 2018; Schmidt et al., 2004; Valenta et al., 2018).

Associated with fruit colour as a long‐range cue, the preference of fruits by a frugivore has also been shown to be dictated by the sucrose and hexa‐sugars in the fruit (Ko et al., 1998; Riba‐Hernández et al., 2005). Some passerine birds avoid fruits with high sucrose content because of their inability to assimilate sucrose efficiently. A review by Lotz and Schondube (2006) showed that omnivorous birds in the Furnariidae (cincodes), Muscicapidae (robins), Mimidae (mockingbirds), Turtidae (thrushes), Sturnidae (starlings) families, lack sucrase and are unable to assimilate sucrose altogether. Amongst the frugivores, those in the Pycnonotidae (bulbuls) and Bombycillidae (waxwings) families have relatively poorer sucrose assimilation efficiencies. In contrast, mammal‐dispersed fruits often contain high concentration of sucrose, compared with glucose/fructose (Martínez del Rio & Restrepo, 1993).

Many of the studies on fruit colour and size, and frugivore interactions have been from the neotropics (Valenta & Nevo, 2020), and less from tropical Asia where information on frugivory and seed dispersal remains patchy (Corlett, 2017). Due to biogeographical differences, the hypotheses that have been developed may not be completely applicable in the old world tropics (Valenta & Nevo, 2020). For example, Onstein et al. (2017) showed that small‐seeded (<4 cm) and understorey palms have smaller dispersal distance and faster speciation rates because they are typically dispersed by small‐bodies frugivores with restricted dispersal range. However, this does not completely hold true in the old world rainforests, in view of the paucity of small‐bodied, sedentary understorey frugivores such as birds.

Palms (Arecaceae) are a species‐rich animal dispersed plant family with dominant species assemblages in tropical rainforests (Couvreur et al., 2011). A large number of bird and mammal frugivores has been reported feeding on palm fruits (Zona & Henderson, 1989). Consequently, several key studies focusing on palms have been undertaken to understand the selective drivers on plant–frugivore interactions including the dispersal syndrome hypothesis. In a classic study, Galetti et al. (2013) showed that areas deprived of large avian frugivores for several decades have smaller palm seeds than non‐defaunated areas. More recently, it has been demonstrated that global variation in palm fruit size was better explained by present‐day frugivore assemblage than by Late Pleistocene assemblages. This is so with the exception of the neotropics, where some large‐palm fruited species may have outlived their main seed dispersers (Lim et al., 2020). Palm fruits that are small (<2 cm) and are brightly coloured also tend to be bird dispersed, whereas those that have larger and dull‐coloured fruits tend to be dispersed by large bodied frugivores including extinct mega‐fauna (Nascimento et al., 2020). Mirroring the spread of plant dispersal‐frugivory studies globally, to our knowledge, studies on the dispersal and frugivory of palms in tropical Asia are rare.

*Licuala ferruginea* is an understorey palm that occurs in the lowland rainforest of Peninsular Malaysia, Singapore and Sumatra in Indonesia. It is relatively common and can dominate the understorey flora where it is found in Peninsular Malaysia (LaFrankie & Saw, 2005). In contrast, *L. ferruginea* is nationally endangered in Singapore, and occurs in scattered small clumps in the rainforest fragments within the island state (Lindsay et al., 2022; Loo et al., 2015). The fruits are drupaceous, crimson to pink in colour when immature and ripening dark purple to black, 15–17 by 12–15 mm when fresh, with a smooth surface. Whilst most other *Licuala* species have fruits that are green when immature and ripen orange to red, *L. ferruginea* and its alliance are one of the few species with immature fruits that are crimson to pink and then ripen black. They are also animal dispersed, most likely by birds and small forest mammals (Saw, 1997; Saw et al., 2003).

As part of a study to understand the dispersal ecology of an endangered understorey palm, *L. ferruginea*, in the rainforest fragments of Singapore, camera traps were deployed at fruiting palms to record the animals that could be predating or dispersing the fruits. Preliminary observations from a fruiting *L. ferruginea* palm at the Bukit Timah Nature Reserve (BTNR) in 2020 were reported in Loo et al. (2020). The observations showed that the fruits were left untouched by the Emerald Dove (*Chalcophaps indica*), Clouded Monitor Lizard (*Varanus nebulosus*) and Common Treeshrew (*Tupaia glis*), but eventually predated by Long‐tailed Macaques (*Macaca fascicularis*). This suggested that the fruits were not successfully dispersed. This monitoring study was expanded to 17 fruiting *L. ferruginea* palms at BTNR, Central Catchment Nature Reserve (CCNR) and Singapore Botanic Gardens (SBG) in 2021 and 2022. To fully document the fruiting phenology of *L. ferruginea*, two additional fruiting palms grown in the nursery were monitored over a period of time. This was necessary as fruits of the palms were subject to a high level of predation in the wild, thereby making it difficult to document the complete stages of fruiting.

In this study, we first describe the gradation in *L. ferruginea* fruit colour and then investigated how the change in colour could be an important cue to animal dispersers. Specifically, we ask the following questions: (a) Does the gradation in fruit colour correspond to the variation in light reflectance and glucose‐sucrose content associated with colour cues to particular animal dispersers? (b) What animal dispersers have been observed at the camera traps taking the fruit, and at which stage of fruiting? From both questions (a) and (b), we then sought to identify the most likely animal disperser of *L. ferruginea* fruits in the rainforest fragments. While this is a small‐sample size study, made challenging by the fact that the palms do not fruit every year, we believe that our observations and analysis can contribute to our understanding of the persistence of endangered understorey palms in rainforest fragments where the loss of frugivores could be prevalent.

## 2 MATERIALS AND METHODS

### 2.1 Study overview

**FIGURE 1:**
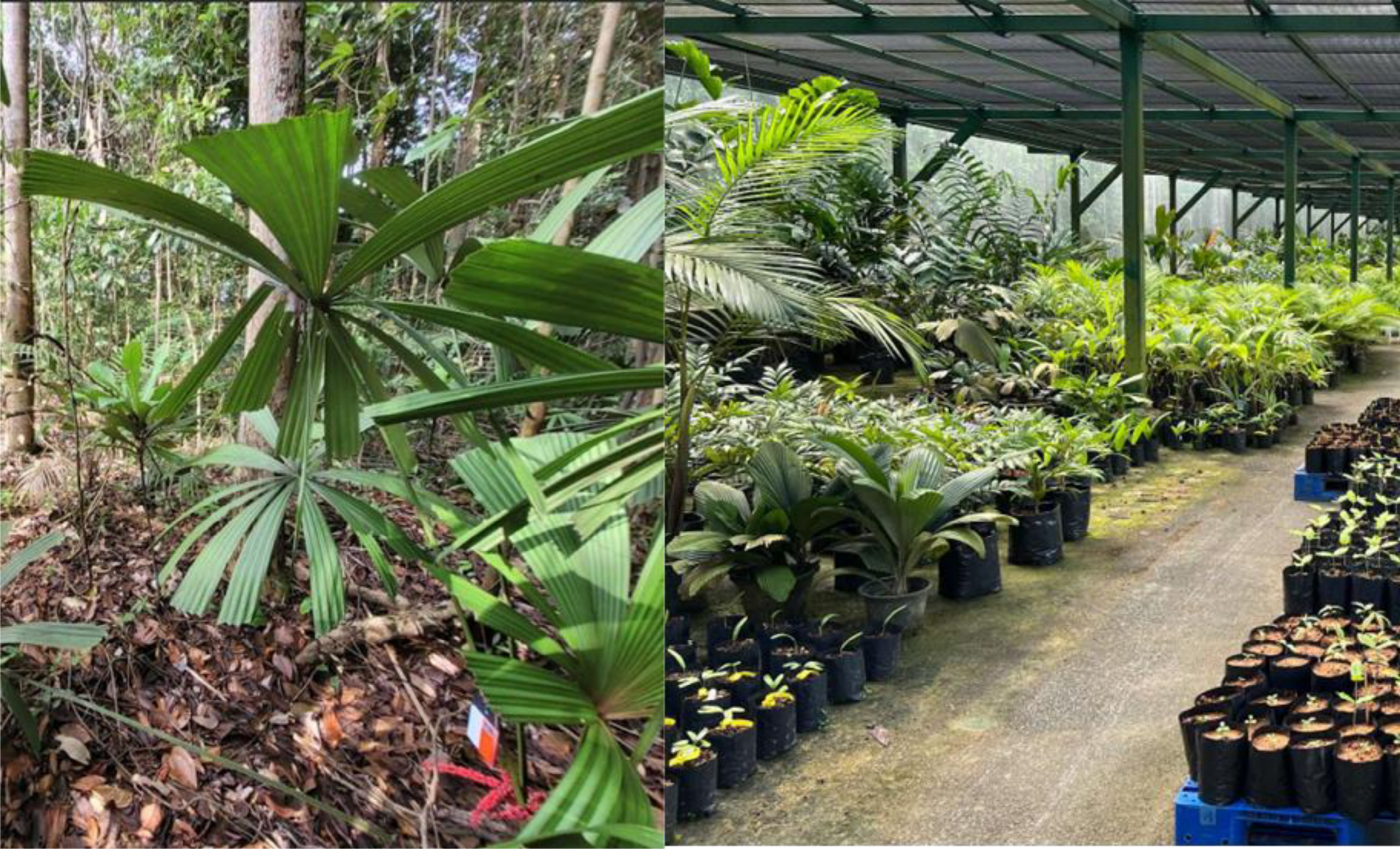
(Left) Natural lowland primary and secondary rainforest fragments of Singapore (Right) Growing shed in nursery with understory‐lighting conditions in Pasir Panjang Nursery (PPN).

**FIGURE 2:**
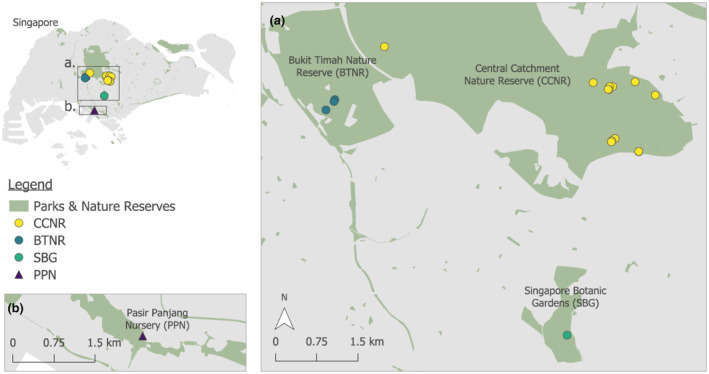
I–Geographical locations of different study sites under (a) natural lowland primary and secondary rainforest fragments of Singapore and (b) growing shed in nursery with understorey‐lighting conditions. II–Zoomed‐in geographical locations of sites under environment (a) (Bukit Timah Nature Reserve [BTNR], Central Catchment Nature Reserve [CCN] and Singapore Botanic Gardens rainforest [SBG]). III‐Zoomed‐in geographical location of site under environment (b) (Pasir Panjang Nursery [PPN]).

This study was conducted in the field and nursery as follows: (a) natural lowland primary and secondary rainforest fragments of Singapore and (b) growing plant house in the nursery with understorey‐lighting conditions (Figure 1). Within the lowland rainforest fragments at Bukit Timah Nature Reserve (BTNR; between 1.3463 and 1.3627 latitude and 103.7695 and 103.7876 longitude), Central Catchment Nature Reserve (CCNR; between 1.3355 and 1.4157 latitude and 103.7741 and 103.8380 longitude) and Singapore Botanic Gardens Rainforest (SBG; between 1.3111 and 1.3149 latitude and 103.8151 and 103.8170 longitude), clusters of *L. ferruginea* palms were identified (Figure 2 I–II). A total of 17 fruiting *L. ferruginea* palms were identified and monitored over 2 years, from 2021 to 2022, using camera traps. This sought to identify animals that were visiting and feeding on the fruits, and hence understand the frugivores that were dispersing the fruits of *L. ferruginea* palms.

At the same time, two individual *L. ferruginea* palms collected from the wild were grown at the Pasir Panjang Nursery (PPN) in Singapore under partial shade to mimic understorey lighting conditions of natural habitat (Figure 2 I and III). The two palms were monitored continuously and their fruiting phenology were documented to gain insight into fruit colour and biochemical changes (i.e. sugar content) associated with the different stages of fruit development. This was necessary as it was difficult to monitor the complete fruiting phenology of the *L. ferruginea* palms in the wild due to intense predation pressure on the fruits. In the nursery, 16–16‐16 NPK fertiliser was applied once every 2 weeks to the palms, thereby providing sufficient required nutrients to enable fruiting all year round, as opposed to one or fewer fruiting cycles per year as observed in the wild.

### 2.2 Capturing animal visitation and feeding of fruiting *Licuala ferruginea* palms via camera traps

A total of 17 camera traps (Bushnell Trophy Cam HD Aggressor and Reconyx Hyperfire HC500) were set up between 5 May 2021 and 20 Jun 2022, one camera trap for each of the identified fruiting *L. ferruginea* palms in the BTNR, CCNR and SBG. Each camera trap was mounted 30–50 cm above ground level and facing the infructescences of the fruiting palm, and left in the field until there were no longer any fruits. This resulted in a total accumulated effort of 1270 camera trap nights. For each animal appearance that triggered the camera traps, a 10‐to‐15‐second video was captured and later analysed to identify the animal. Observed animal behaviours in each captured video were categorised into two activities: (1) feeding, and (2) visiting, which refers to animals that were captured in the camera trap, but were not observed to be feeding on the fruit. Captured videos of the same animal with interval less than 15 min were considered as replicates and counted as one independent capture. In addition to the animal visitation and feeding data collected from 17 *L. ferruginea* palms in the wild, additional opportunistic observations were also made on the two *L. ferruginea* palms cultivated from seed in PPN.

### 2.3 Germination of seeds from *Licuala ferruginea* fruits

**FIGURE 3:**
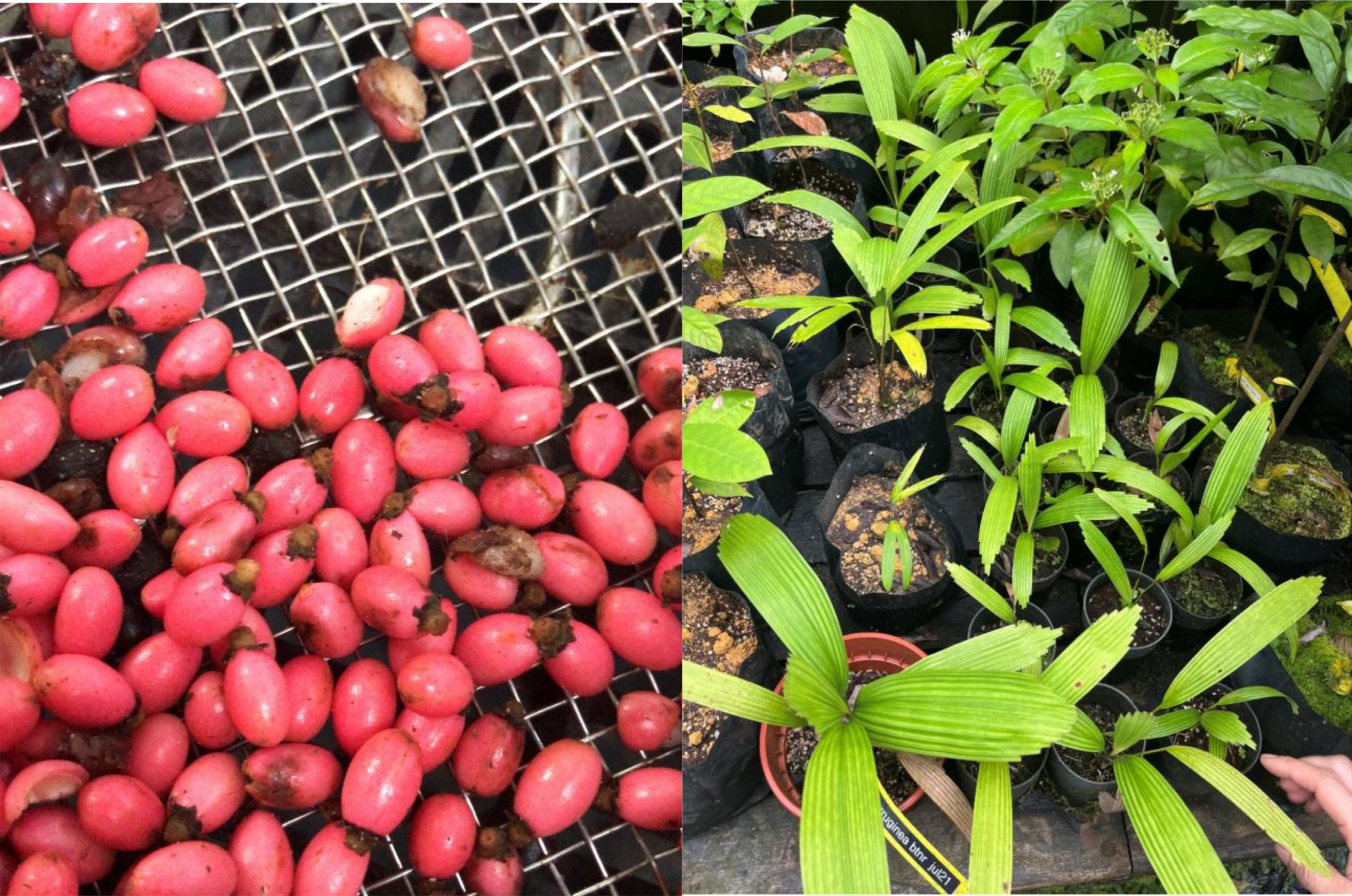
(Left) Fruits at the pink stage which were cleaned and seeds sown in a nursery; (Right) Seedlings grown from the collected seeds.

Fruits (Figure 3) from a single *L. ferruginea* individual in Bukit Timah Nature Reserve were collected opportunistically from an infructescence which became detached from the parent plant. The flesh of the fruits was removed manually by peeling and washing, and the seeds sown in perlite and cocopeat media in a nursery.

### 2.4 Documentation of fruiting phenology, spectral study and chemical testing of two *Licuala ferruginea* palms grown in Pasir Panjang nursery

#### 2.4.1 Photo documentation

**FIGURE 4:**
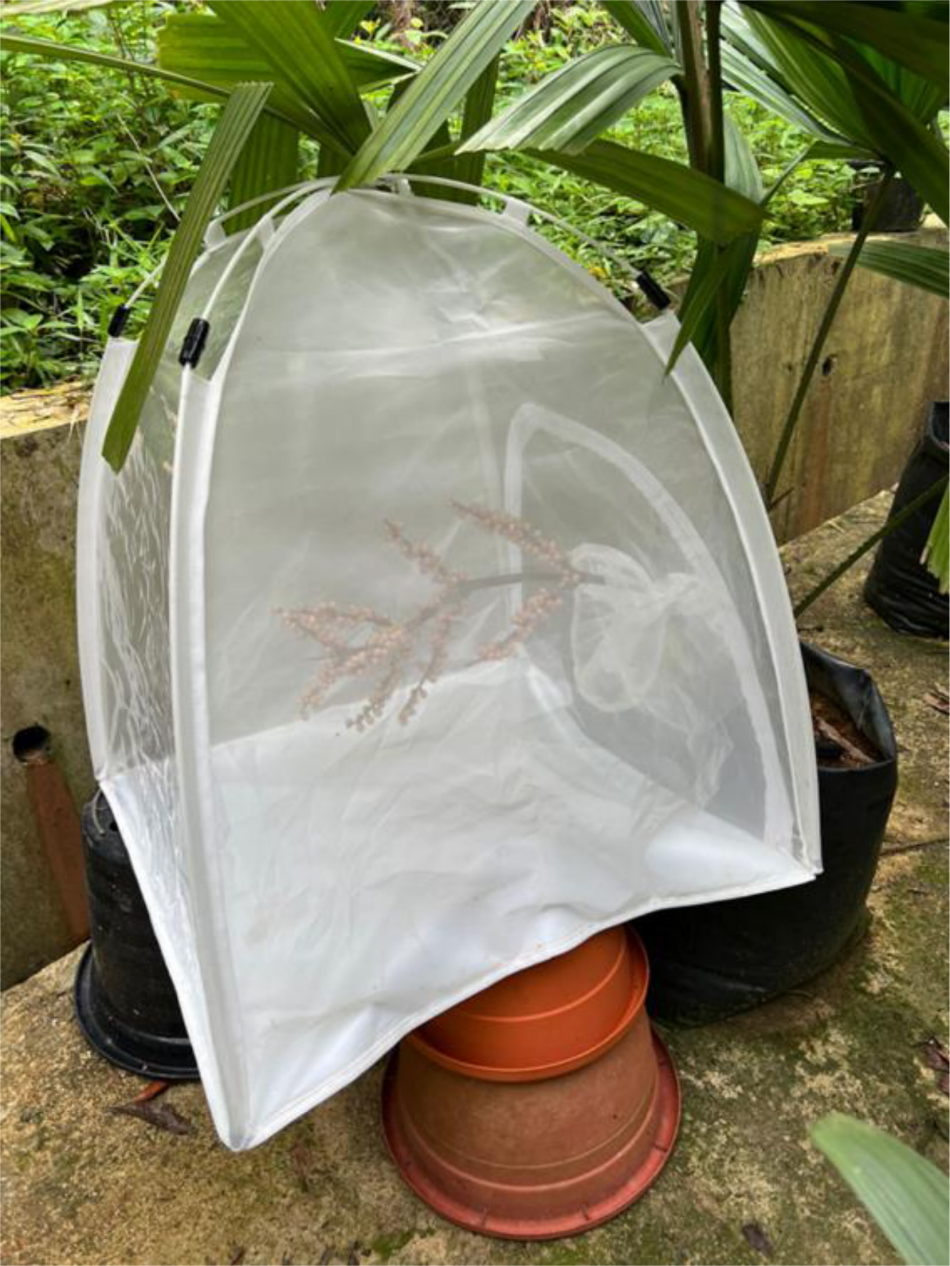
Developing *Licuala ferruginea* fruits encased in Bugdorm‐2120 insect rearing tent to protect them from being predated by frugivores.

The two *L. ferruginea* palms in the PPN were checked closely for any indication of flowering. As soon as the flower buds emerged, the palms were monitored weekly. RGB photos were taken weekly for the same palms from the same angle to document flowering and fruit development. When fruits were set, Bugdorm‐2120 insect rearing tents were used to protect developing fruit bunches from being predated by frugivores (Figure 4).

#### 2.4.2 Sampling of fruits

One bunch of fruits from each of the *L. ferruginea* palms was randomly selected on the 4th, 5th, 8th, 9th, 13th, 14th, 15th, 16th and 17th weeks after fruit set, for quantitative measurement of fruit sizes, spectral reflectance and sugar content. Due to limited number of fruit bunches produced by two *L. ferruginea* palms, only up to a total of three fruit bunches were sampled from each palm per fruiting cycle. The data over several fruiting cycles were pooled to obtain full dataset for complete fruit development with nine time points as indicated formerly.

#### 2.4.3 Fruit size measurement

Collected fruits were measured to obtain width and length of fruits, which were then plotted using *ggplot* and *geom_smooth* in RStudio 2021.09.0 build 351 to study the changes of fruit length and width over time.

#### 2.4.4 Colour of fruits

##### Relative reflectance UV–VIS range (250–700 nm)

Relative reflectance spectra in 250–700 nm range of collected fruits were measured using Ocean Insight Flame Miniature Spectrometer with attached Ocean Insight PX‐2 Pulsed Xenon Light Source positioned at 45° of fruit surface. Average relative reflectance spectra for all fruits were calculated using Rstudio 2021.09.0 build 351.

##### Relative reflectance in VIS–NIR range (400–900 nm)

Reflectance spectra in 400–900 nm range of the fruits attached on the individual palms were measured weekly using the handheld SPECIM IQ device from the 4th week until 20th week after fruit set. Pixel of fruits in collected his images were labelled using Scyven (Scyllarus Visualisation Environment) developed by National ICT Australia Limited (NICTA). Relative reflectance spectra of all labelled fruit pixels were extracted. Average relative reflectance spectra for all fruits were calculated using Rstudio 2021.09.0 build 351.

#### 2.4.5 Qualitative colour change in response to pH

Anthocyanin has been reported to be the main colour pigment responsible for colour of many palm fruit species (Hazir et al., 2012; Martín et al., 2017). To further confirm the nature of colour pigments of *L. ferruginea*, pigments of collected ripe fruits were extracted using acidified ethanol (400 mL 92% ethanol: 75 mL acetic acid), with incubation time of 4 hours (Silva et al., 2019). Glacial acetic acid and 10% KOH solution were added separately to sample solutions of extracted pigments. RGB photos of colour of pigment solutions in different pH conditions were taken.

#### 2.4.6 Fruit nutrient content

For each event of fruit sampling, flesh of collected fruits were separated from seeds and combined to make three 5‐gram composite samples. The samples were sent to BV‐AQ (Singapore) laboratory for testing of hexoses (fructose, galactose, and glucose), disaccharides (lactose anhydrous, maltose, and sucrose) and total sugar using gas chromatography.

## 3 RESULTS

### 3.1 *Licuala ferruginea* fruiting phenology and fruit properties

#### 3.1.1 Flowering and fruit development

**FIGURE 5:**
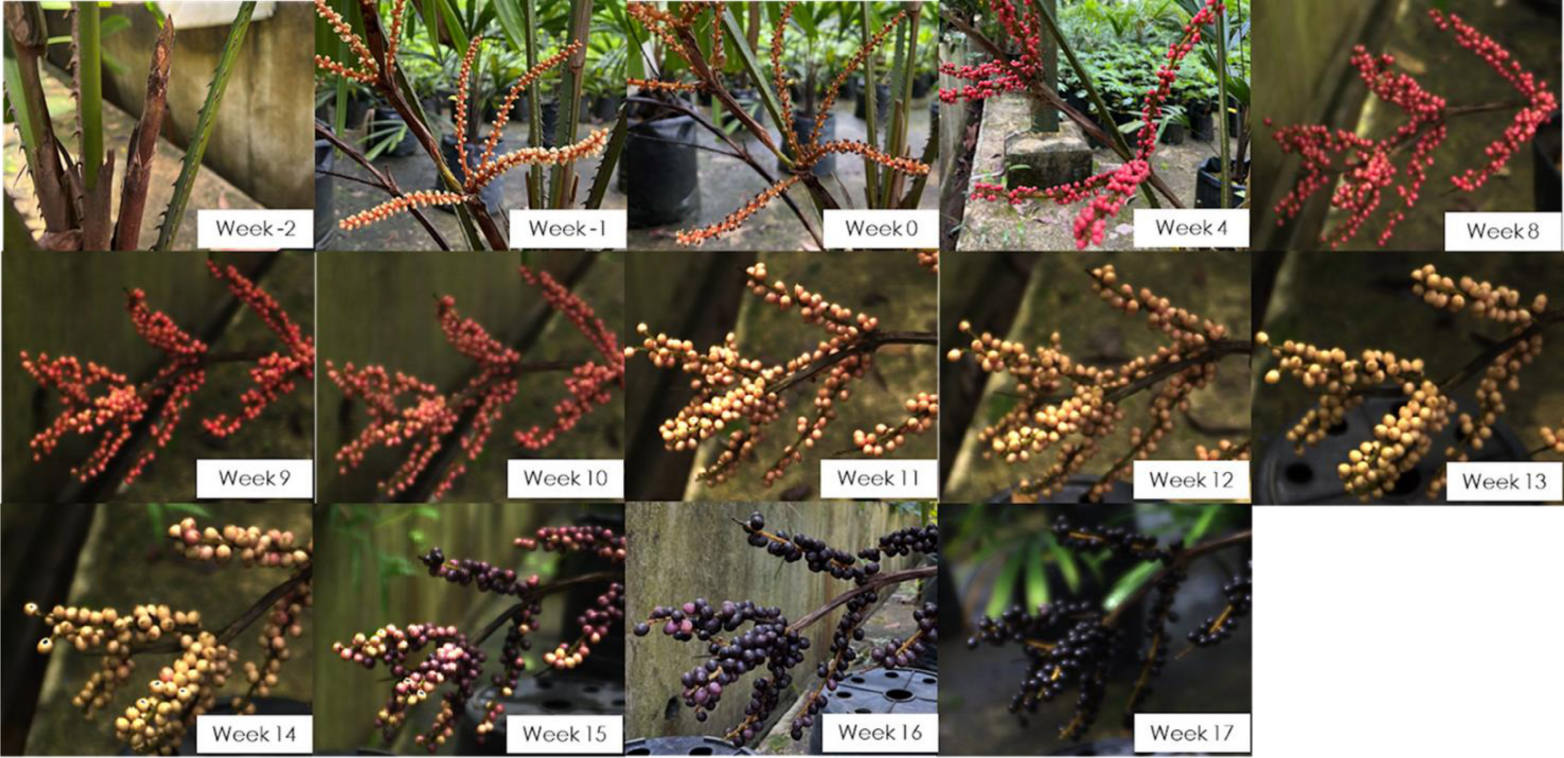
Flowering and *Licuala ferruginea* fruit development. Fruit set was marked as week 0 for the purpose of tracking *L. ferruginea* fruit development.

**FIGURE 6:**
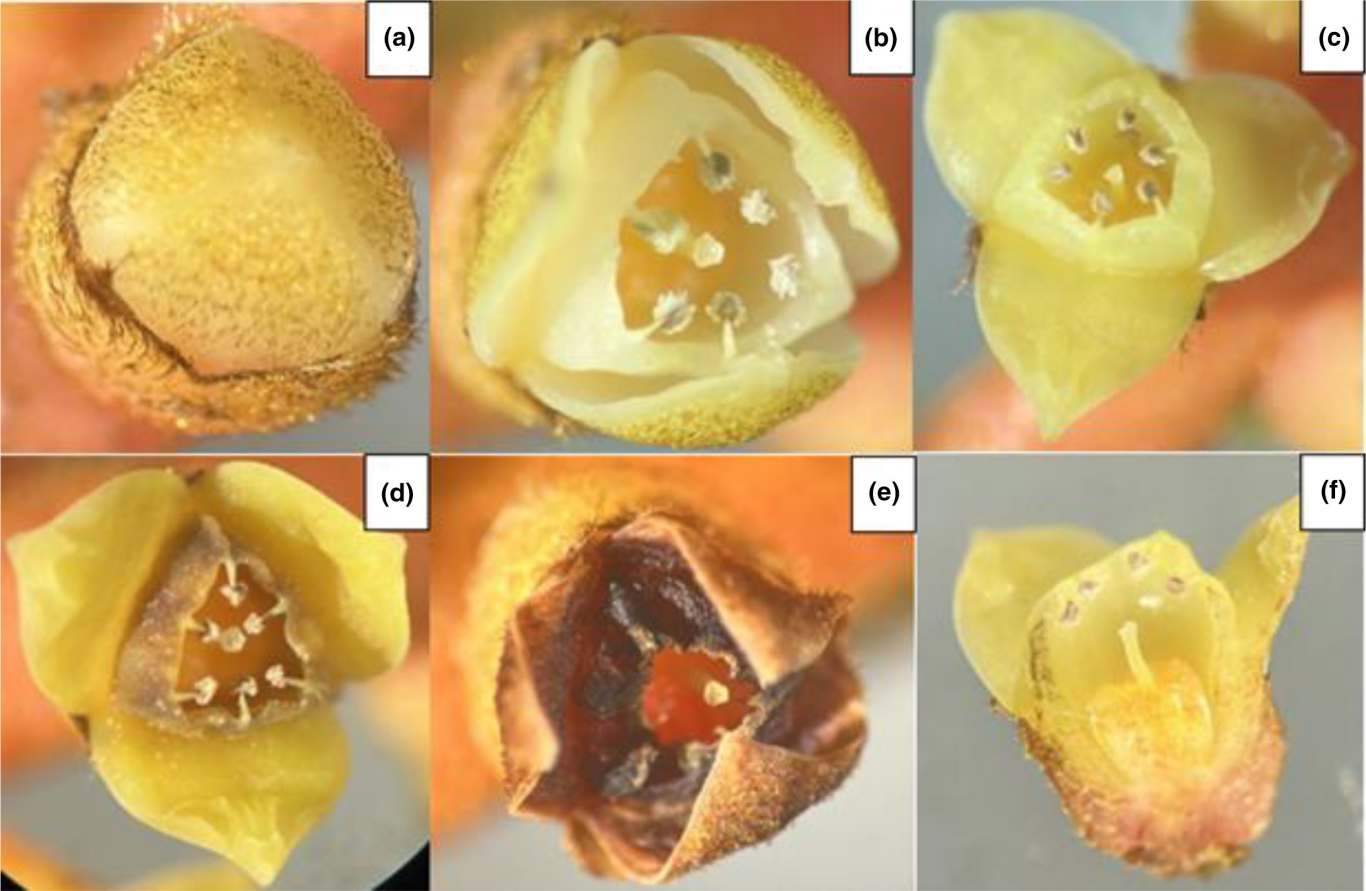
(a–e) Flowering phenology of *Licuala ferruginea* from bud to after flower is pollinated. (f) Cross‐section of blooming *L. ferruginea* flower.

In contrast to the wild where *L. ferruginea* palms were observed to be irregular in fruiting and did not fruit yearly, the *L. ferruginea* palms grown in the nursery fruited all year round, with ~2‐month intervals between fruiting cycles. A complete flowering and fruiting cycle lasted for 6 months starting from emergence of flower buds to fruit ripening. The fruiting cycle for the *L. ferruginea* palms in the nursery started with inflorescences emerging from the base of palms (Figure 5 – Week −2). Inflorescences rapidly elongated and flowers opened within 1 week (Figure 5–Week −1). LF flowers had three petals and stigma positioned below 6 anthers (Figure 6). Flowers lasted for a week, dried up upon pollination (Figure 6) and fell off (Figure 5–Week 0). The two cultivated LF individuals were isolated and did not have any nearby *L. ferruginea* palms. Their flowering and fruiting cycles did not overlap. The fruit set rates for both individuals were observed to be ~70%.

Within 4 weeks after fruit set, bright red fruits were observed. The bright red colour of fruits remained unchanged until Week 8 after fruit set, when the colour gradually faded to white by Week 13 (Figure 5–Week 9–13). Fruits then changed gradually from white to dark purple by Week 15 (Figure 5–Week 15). The colour darkened to black from Week 17 onwards (Figure 5–Week 17). At this stage, fruits were considered ripened as the fruits became soft with fluffy pulp, as opposed to being firm in previous stages. After Week 17, fruit colour remained unchanged, with no further visible changes to fruit morphology. If not eaten by frugivores, fruits eventually dried off and fell onto the ground. The fruits were not scented at any stage of development.

Given the described colour changes, LF fruit colour development could be categorised into five main stages: (I) Red, (II) Pink, (III) White, (IV) Purple and (V) Black. These various stages of fruit colour could also be seen in the wild *L. ferruginea* palms that were being studied in BTNR, CCNR and SBG. This colour change to white has not been described previously. Saw (1997) recorded that the fruits are ‘crimson to pink ripening dark purple to black’.

#### 3.1.2 Fruit size

**FIGURE 7:**
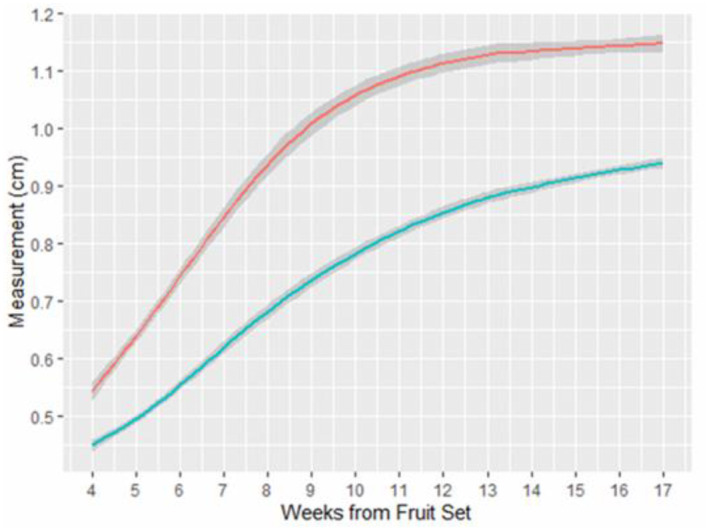
Average measurement of LF fruit width (blue) and length (red) over time. Graph was obtained using ggplot and geom_smooth in RStudio 2021.09.0 build 351.

The length of fruits showed a linear increase from 0.580 ± 0.009 cm on Week 4–0.870 ± 0.015 cm on week 8 (Figure 7). From Week 8, increase in fruit length started to slow down and reached averaged max values (~1.15 cm) from Week 13 onwards. Similarly, fruit width showed a relatively linear increase for the first 8 weeks and slowed down from Week 8–Week 17 (Figure 7). Fruit width doubled from 0.470 ± 0.006 cm on Week 4 to 0.940 ± 0.006 cm on Week 17. Compared with the fruit dimensions (15–17 mm by 12–15 mm) given in Saw et al. (2003), the *L. ferruginea* fruit sizes in our study were smaller.

#### 3.1.3 Fruit relative reflectance

**FIGURE 8:**
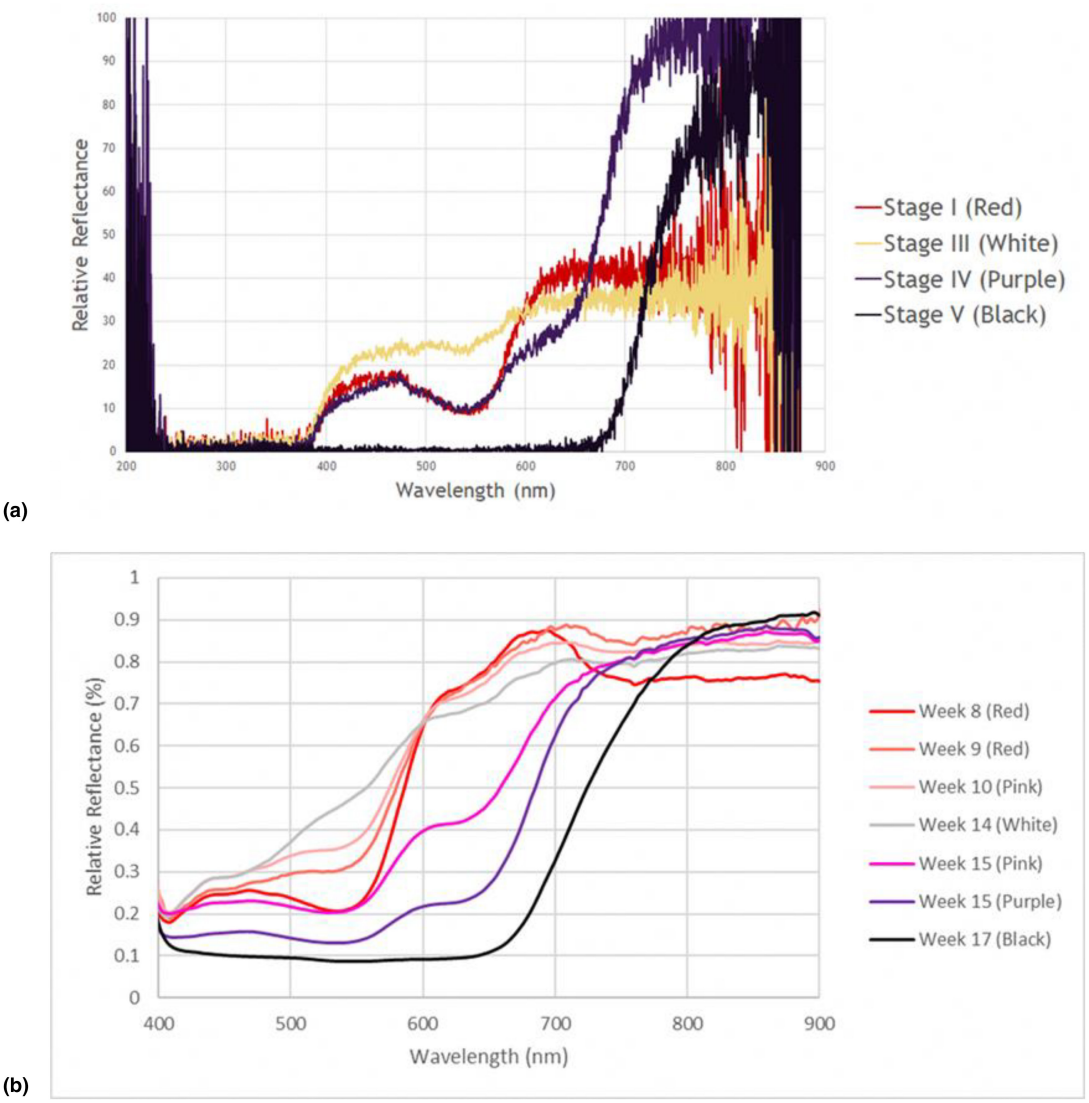
Relative reflectance of LF fruits at different stages of development obtained by: (a) Ocean Insight Flame Miniature Spectrometer with attached Ocean Insight PX‐2 Pulsed Xenon Light Source positioned at 45° of fruit surface; (b) handheld SPECIM IQ device.

As seen from the relative reflectance graphs for fruits at different stages (Figure 8a,b), *L. ferruginea* fruits did not reflect light in the ultraviolet range. Pigments responsible for red colour in Stage I absorbed more green region (550 nm), and reflected more blue region (450 nm) and red region (600 nm) wavelengths. The peak relative reflectance for red light was two to three times as high as that for blue light, leading to the red colour perception of the pigments.

As fruits faded from red to white in Stage III, the relative reflectance for blue region (450 nm) and red region (600 nm) remained relatively unchanged (Figure 8b). Instead, the relative reflectance in green region increased as fruits faded to pink in Stage II and white in Stage III of development. When fruits turned purple in Stage IV, relative reflectance of red and green regions decreased significantly, while that of blue region also remained relatively constant. Since relative reflectance in the red region was not as intense as Stages I to III, Stage IV fruits appeared purple. As the fruits darkened further to black in Stage V, all wavelengths within the visible spectrum (380–700 nm) were almost completely absorbed by fruit pigments. Additionally, the relative reflectance of pigments in Stage IV and Stage V in NIR region were two times as high as that of pigments in Stage I and Stage III (Figure 8a).

#### 3.1.4 Fruit colour change in response to pH

**FIGURE 9:**
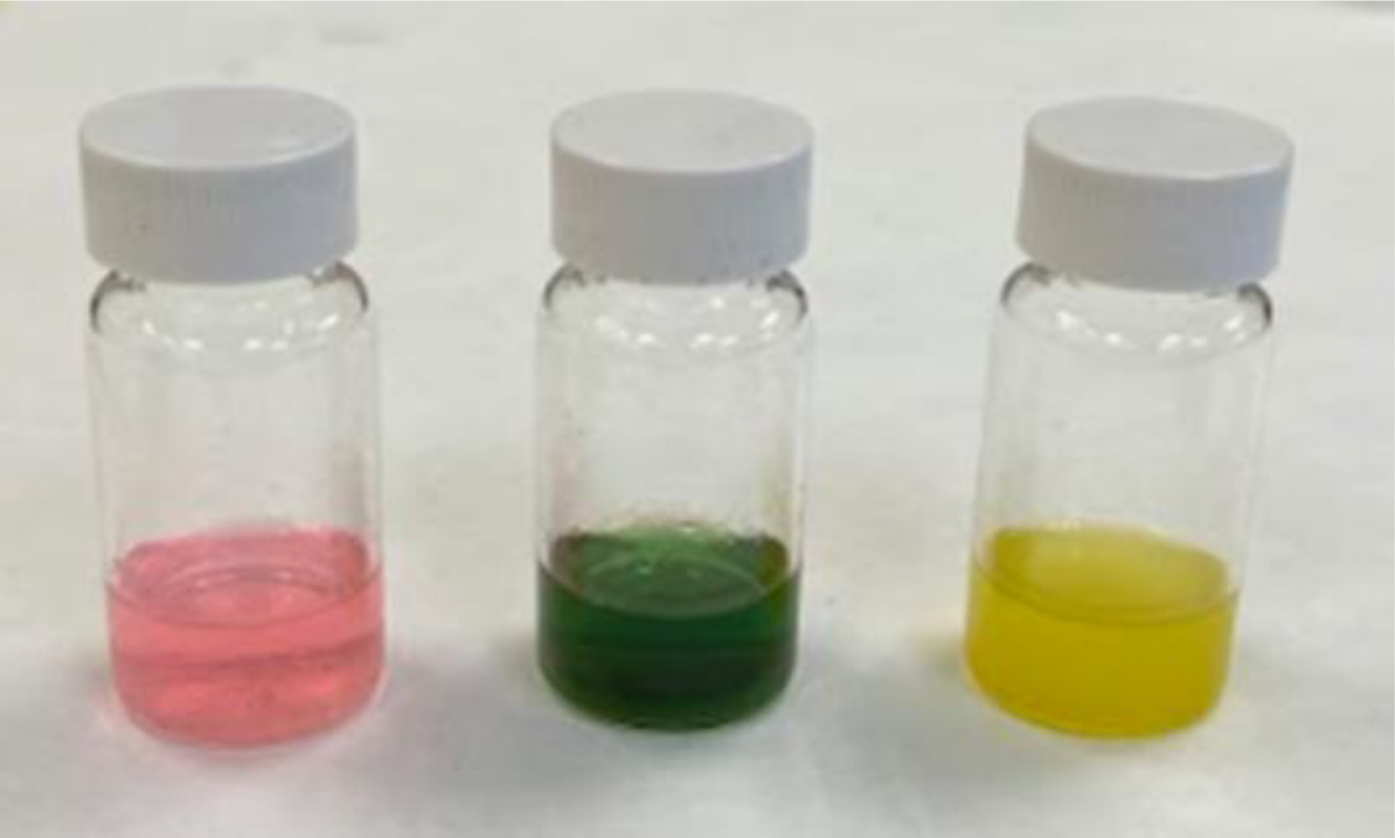
Colour of pigments extracted from stage V LF fruits by acidified ethanol in different pH conditions. Left‐Glacial acetic acid (acidic environment); Middle‐10% KOH solution (alkaline environment); Right‐10% KOH solution for extended period of time.

Pigments extracted from Stage V fruits were pink or red in acidic solution and green in alkaline solution (Figure 9). These colour changes were reversible by changing pH of the solution. When left in 10% KOH solution for an extended period of time, the green colour of extracted pigments slowly changed into yellow (Figure 9). The described colour change was not reversible, suggesting pigment degradation. The observed pH‐dependent colour changes resemble that of anthocyanin pigments including those reported in palms (Ananga et al., 2013; Singh et al., 2014), thereby inferring that the fruit colour changes in *L. ferruginea* palms are brought about by anthocyanin pigments.

#### 3.1.5 Fruit nutrient content

**FIGURE 10:**
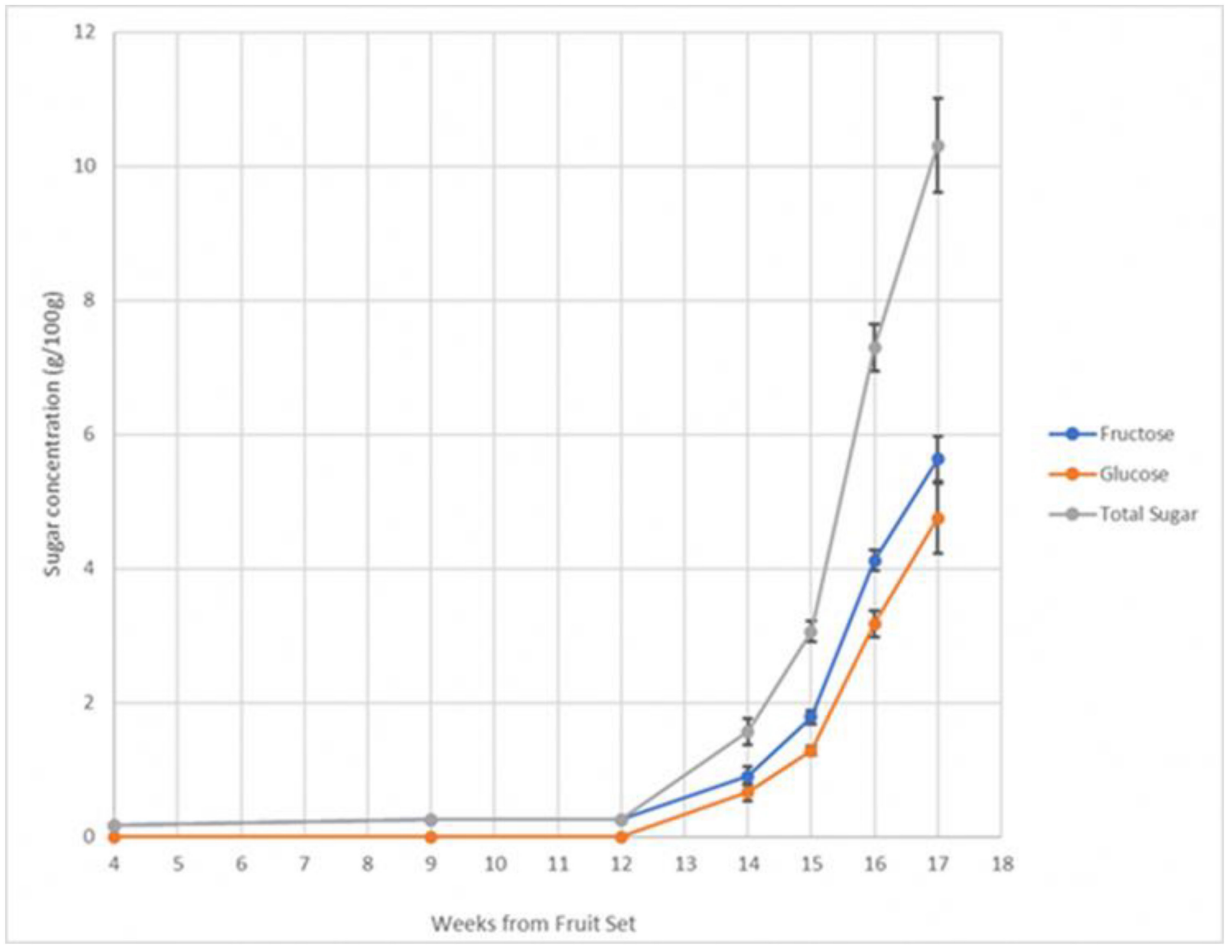
Concentrations of detectable sugar over time after fruit set.

Of all the tested hexoses and disaccharides, only fructose and glucose were detected. For the first 13 weeks of fruit development, only traces of fructose were detected in the fruits. Levels of fructose and glucose started to increase rapidly from Week 14 onwards as the fruits turned dark purple and then black, suggesting that they were ripe (Figure 10).

### 3.2 Germination of seeds from pink *Licuala ferruginea* fruits

Seeds obtained from fruits at ~10 weeks after fruit set had a hard endocarp and germinated under nursery conditions after several months (Figure 3). This showed that seeds at this stage would likely be able to germinate in field conditions.

### 3.3 Frugivore visitation and feeding study via camera trapping

**FIGURE 11:**
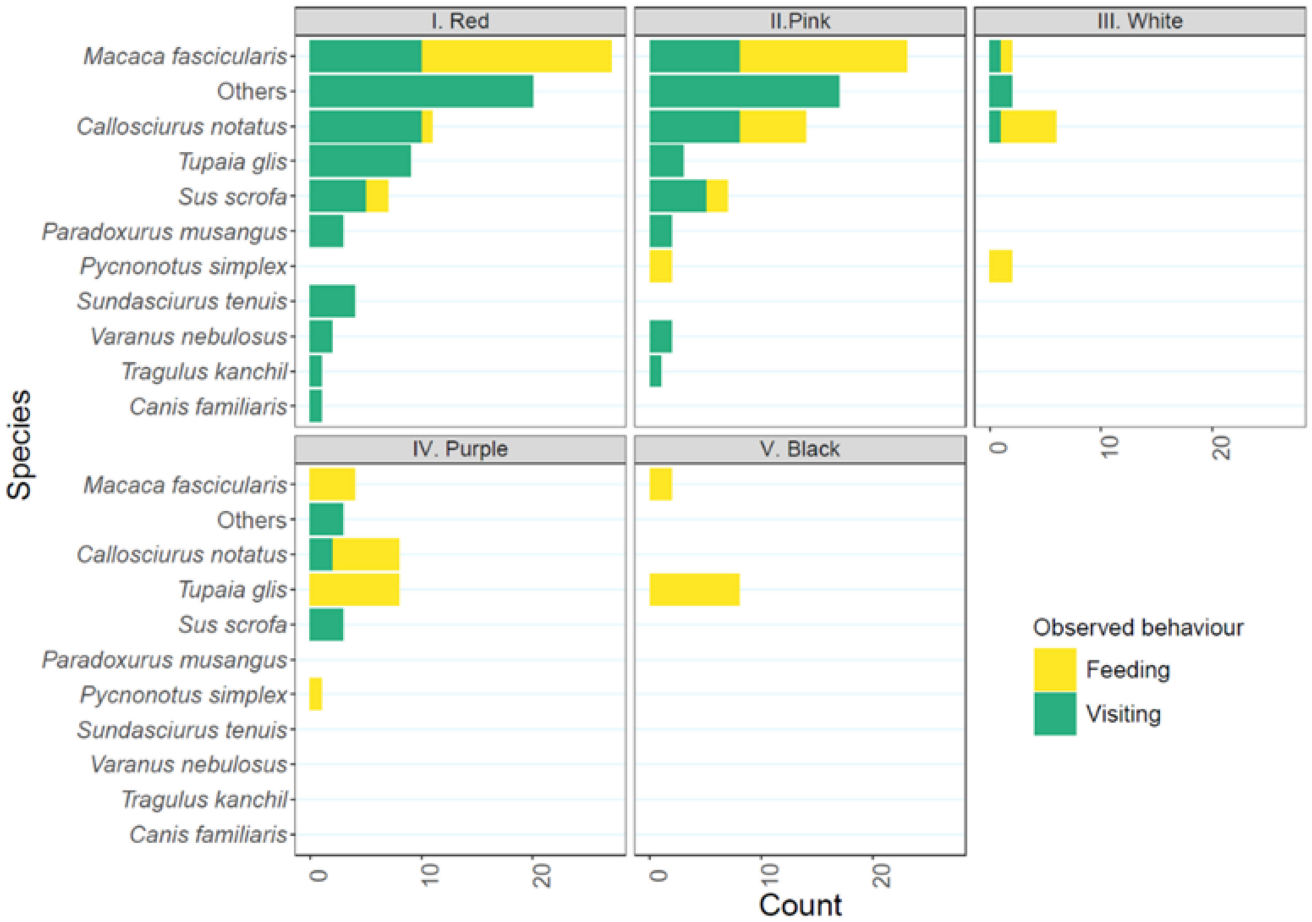
The breakdown on the recorded species and its observed behaviour in the camera traps.

A total of 2395 camera trap videos were processed from the camera traps, of which 333 videos (13.9%) were independent captures. Ten species were identified (Figure 11), the majority of which were mammals. The Long‐tailed Macaque was the most common species that appeared in 655 videos (27.3%) and was found to be the most common species visiting and eating the palms when they were fruiting. They showed higher visitation rates when the fruits were red or pink in colour (i.e. Stages I and II). The next most numerous species were the Wild Boar (*Sus scrofa*), appearing in 198 videos (8.2%), and the Plantain Squirrel (*Callosciurus notatus*), appearing in 195 videos (8.1%). Both species were also recorded feeding mainly when fruits were red or pink in colour (i.e. Stages I and II).

**FIGURE 12:**
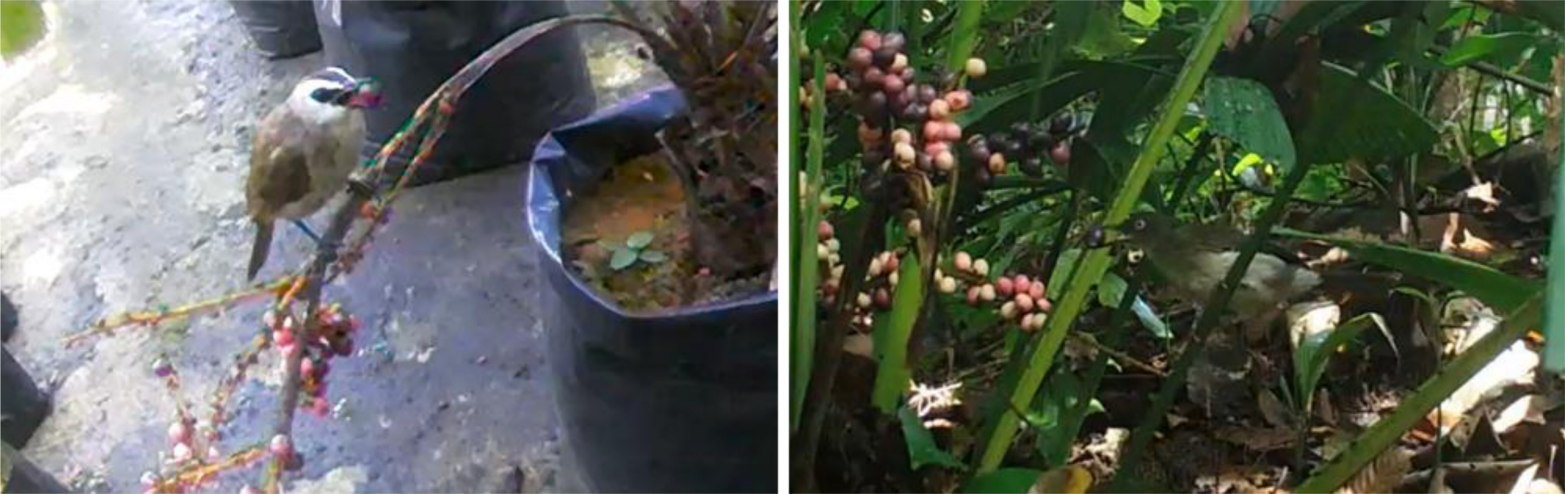
Screen captures of camera videos showing a yellow‐vented bulbul (left) and a cream‐vented bulbul (right) feeding on a purple fruit.

A Cream‐vented Bulbul was observed visiting a palm with full‐sized pink, white and purple fruits and plucking a purple fruit at BTNR, before flying away with the fruit in its beak (Figure 12, right and Video 1). This was the only bird that was observed feeding on the mature fruit of *L. ferruginea* in the wild.

**VIDEO 1:** Cream‐vented bulbul with a purple *Licuala ferruginea* fruit from a palm in BTNR.

At the PPN site, a Yellow‐vented Bulbul (*Pycnonotus goiavier*), a common species of parkland, scrubland and gardens in Singapore, was found feeding on purple and black fruits of one of the palms (Figure 12, left).

## 4 DISCUSSION

In this study, we observed that *L. ferruginea* fruits exhibit five different stages of colour as the fruits develop: red when unripe to pink, white, and then purple and black when ripe. Tests on extracted pigments from ripe fruits showed that anthocyanin pigments were responsible for the colour changes. In flowering plants, anthocyanin pigments play major roles in (1) protection of plant cells against harmful free radicals from oxidative stress caused by various environmental factors (i.e. UV radiation under intense sun light, heat, drought, pollution) and (2) frugivore attraction through contrasting fruit colouring to aid seed dispersal (Pervaiz et al., 2017). In the context of *L. ferruginea* palm fruits, protection against oxidative stress is redundant because this species occurs in the understorey of lowland tropical rainforest where light intensity is low, precipitation is abundant and temperature is relatively stable year‐round (Willson & Whelan, 1990). That means biosynthesis of anthocyanin in *L. ferruginea* fruit is likely for the purpose of frugivore attraction.

Given the considerably high energy cost of anthocyanin biosynthesis (Cerqueira et al., 2023), why would *L. ferruginea* palms invest in this? Interestingly, this anthocyanin‐centric fruit colouring adaptation is not just unique to *L. ferruginea*, but has been observed across various understorey fruiting plant species, especially in tropical forests (Galetti et al., 2003). It has been found that anthocyanin compounds display a wider array of contrasting colours from black, purple, blue to pink, red and even white, compared with other simpler biochemical structure and less costly to produce pigments like carotenoid that produce colours within the same hue (i.e. red, orange and yellow) (Kapoor et al., 2022). Furthermore, as demonstrated in this study, colour change of anthocyanins can be accomplished by simple change of pH, indicating the versatility of the pigments as a colouring agent (Figure 9). Perhaps, the range of fruit colours offered by anthocyanin as visual cues to attract frugivores for seed dispersal outweighs the higher energy investment in the pigment. This is particularly so in equatorial tropical forests that lacks a cool season restricting growth, and fruiting phenological cycles are more varied with some species reproducing once in several years (Sakai & Kitajima, 2018). This coupled by the dense rainforest canopy, may result in plants that fruit irregularly, having to invest in a wider range of fruit colours to attract frugivores. This could certainly be the case for the understorey *L. ferruginea* palms, which tends to fruit irregularly in the wild, as opposed to the regular fruiting of the two well‐fertilised individual palms in the nursery in this study. Based on this hypothesis, we would then expect fruit colour changes to be visual cues for specific frugivores.

Contrasting fruit colours of red, purple and black are rich in anthocyanins and have been shown to attract birds (Duan et al., 2014; Schaefer, McGraw, & Catoni, 2008; Sinnott‐Armstrong et al., 2021). This corroborates the camera trap records in this study where two frugivorous bird species, the cream‐vented bulbul in the wild and yellow‐vented bulbul in the nursery, were the only animals found to feed on ripe purple and black *L. ferruginea* fruits in whole (i.e. without crushing the fruit and seeds). Based on the available gape size data of native bulbul species in Singapore (8 out of 10 available species) from McFadden et al. (2022), all species have a gape size (>0.98 cm), which is larger than the fruit size of *L. ferruginea* at Week 17 (0.940 ± 0.006 cm) (Appendix A, Table A1). The remaining two species without gape size data are also likely able to swallow the fruit whole based on its beak width measurement. However, only seven species are primary and old secondary native forest species and potential dispersal agents of *L. ferruginea*, including the cream‐vented bulbul observed in this study. Most of these species are also uncommon to rare in occurrence (Lim et al., 2021), which may explain why there was only one record of the cream‐vented bulbul in the camera traps.

However, this begs the question of the advantage that a red fruit would confer in the initial phase of the *L. ferruginea* fruit development, when it is still unripe and unlikely for any bird to feed on the fruit. Further, why would the fruit then turn pink/white, and finally present itself as purple/black when ripe? Here, we offer a plausible explanation for this.

Based on the spectral reflectance and glucose/fructose content of the fruits obtained at different colour stages, it would appear that the initial phase of red fruits, with high contrasting red reflectance against a green foliage background, might be intended as a form of early advertisement to birds, or may draw attention to adjacent infructescences with purple or black fruits (Lomáscolo & Schaefer, 2010; Melo et al., 2011; Nevo et al., 2018). Willson and Thompson (1982) found some evidence of this in North America, where some plants have unripe red fruits as an early signal of fruiting to birds that occur in low abundance or are migratory in nature. In this case, while the eight forest bulbul species found in Singapore (Table A1) may not be uncommon in the original forest (inferred from larger forest tracts in Peninsular Malaysia (Lim et al., 2021)), only three species frequent the understorey for foraging (i.e. Cream‐vented Bulbul, Olive‐winged Bulbul (*Pycnonotus plumosus*)), and Asian Red‐eyed Bulbul (*Pycnonotus brunneus*) (Table A1). This reflects the paucity of understorey avian frugivores in the old‐world forests (Onstein et al., 2017). This coupled with the inconspicuity of *Licuala* plants in the understorey that does not receive much light, may result in the need for palms to advertise their fruits earlier. In addition, the red‐coloured unripe fruits of *L. ferruginea* may provide protection against early‐stage insect herbivory as most insects are unable to see red colour (Schaefer & Schmidt, 2004; Steyn, 2008). This is advantageous as it could help reduce insect predation on the fruits and allow the fruits to develop further. The subsequent stages of low contrasting pink and white fruit colours, as evident from the increased green reflectance, would have reduced the conspicuity of the fruits against the green foliage background to birds and would‐be predators. In turn, this would enhance the chances for fruits to develop further. Alternatively, the evolution of the ripening fruit colour changes may be a physiological or biochemical necessity en route to the final purple/black fruit colour, or result of the phylogenetic history of the palms (Willson & Thompson, 1982). Saw et al. (2003) described that whilst most other *Licuala* species have fruits that are green when immature and ripen orange to red, *L. ferruginea* and its alliance are one of the few species with immature fruits that are crimson to pink and then ripen black.

Seeds were able to germinate from fruits about 10 weeks after fruit set (at the stage II pink stage), though the fruits were still not full‐sized yet (Figure 10). Seeds were surrounded by a hard endocarp at this stage, which would protect the propagules from mechanical damage that may be caused by foraging and feeding by frugivores. This implies that consumption of fruits at this immature stage does not preclude seed dispersal, and that the fruits may have a long period when they are on the plant during which they are viable if dispersed, even as the absence of glucose and small trace of fructose in the initial stages of the fruits' ripening would have made them unattractive for the birds to feed on (Ko et al., 1998; Lotz & Schondube, 2006). Unfortunately, seeds at other stages of fruit development were not tested for their germination, so it is not possible to say at which stage of fruit ripening frugivory could lead to effective seed dispersal, leading to seed germination. Furthermore, the effect of fruit ripening stage on the germination rate of seeds has been show to vary amongst palms species, with species such as *Syagrus romanzoffiana* (Cham.) Glassman showing a lower rate of germination with more mature fruits (Broschat & Donselman, 1987; Oliveira et al., 2015), while species such as *Phoenix roebelenii* O'Brien and *Roystonea regia* (Kunth) O.F.Cook showed a higher germination rate for seeds from more mature fruits (Broschat & Donselman, 1987). The consequences of dispersal of immature seeds on their germination rate require further study.

The change in colour of the fruits to a contrasting purple, followed by black, was accompanied by a sharp increase in the concentration of glucose and fructose. The combined prevalence of a dark purple/black fruit colour and high nutritional content at the final stage of fruit development would provide a strong signal to birds that the fruit was ripe to feed on (Sinnott‐Armstrong et al., 2020). The lack of scent of the *L. ferruginea* fruits further supports they hypothesis that the fruits are more adapted for bird than mammal dispersal, as it is thought that birds primarily use visual stimuli, rather than olfactory stimuli, to detect fruits (Rodríguez et al., 2013). In contrast, most frugivorous mammals rely on olfactory stimuli to detect ripe fruits, with dichromatic mammals possibly relying more heavily on olfactory than visual stimuli to detect ripe fruits.

While *L. ferruginea*'s fruit colour changes may be targeted at birds, possibly bulbuls in this study, the camera trap record of animals taking the fruits was dominated by the Long‐tailed Macaque and Wild Boar. Both species could be seen visiting and feeding on *L. ferruginea* fruits across the five fruit colour stages. Interestingly, while the Long‐tailed Macaque has trichromatic vision and is capable of differentiating across the 5 fruit colour stages, it fed on red and pink fruits. This could be explained by the fact that red fruits are, to many frugivores, considered more conspicuous when contrasted against foliage and background as compared with relatively more inconspicuous black fruits (Steyn, 2008). On the other hand, the Wild Boar has dichromatic vision and associated red‐green blindness, and hence may not have selected fruits based on their colour stage (Jacobs, 2009; Steyn, 2008). The prevalence of long‐tailed macaques and wild boars in the camera trap records could be attributed to these species being well‐adapted to small forest fragments within an urban landscape where anthropogenic food is readily available (Lamperty et al., 2023; Sha et al., 2009). Their dominance resulted in the destructive feeding of immature *L. ferruginea* fruits, thereby limiting the full development of the fruits and hence dispersal. These observations also suggest the risks of attracting predators or lower‐quality dispersers with increasing conspicuity in unripe fruits (Wheelwright & Janson, 1985), which may have been minimised in larger tracts of original forests where edge species such as the Long‐tailed Macaque and Wild boar are not present in high densities within the forest interior.

The observations of *L. ferruginea* fruit colour changes and bulbuls as the only birds that fed on mature fruits in whole, coupled with gape size matching to the fruit size, provide indicative support for the dispersal syndrome hypothesis, which posits that fruit and frugivores co‐evolve with matching fruit traits (such as colour) and frugivore morphology, behaviour and sensory capacities (Valenta & Nevo, 2020). This study was limited by the small sample of *L. ferruginea* palms that were fruiting in the wild. It was also hampered by the challenge of protecting the immature fruits from being eaten by edge species, such as the Long‐tailed Macaque and Wild boar. Future studies should seek to isolate the effects of these species, so that the contributory role of bulbuls and other frugivorous birds not encountered in this study (e.g. leafbirds) as dispersal agents could be determined. Additionally, studies could test alternative reasons for the production of anthocyanin in plants for *L. ferruginea*, such as its possible role in acting as a repellent against fungal pathogens in the rainforest (Schaefer, Rentzsch, & Breuer, 2008). Findings from this study could also be used as a model to study other understory rainforest plants with a similar colour change of red to purple/black, such as *Hanguana neglecta*. In conclusion, such studies are important in understanding the effects of forest fragmentation on plant–frugivore interactions, including the decline and extirpation of frugivores, for which the regeneration of forest and persistence of plants with small populations could be dependent (Galetti et al., 2006; Lamperty & Brosi, 2022).

## AUTHOR CONTRIBUTIONS

**Kenneth B. H. Er:** Conceptualization (lead); data curation (supporting); formal analysis (supporting); writing – original draft (equal). **Derrick H. D. Nguyen:** Data curation (equal); formal analysis (lead); writing – original draft (equal). **Yi Shuen Yeoh:** Data curation (equal); writing – original draft (equal). **Max D. Y. Khoo:** Data curation (equal); formal analysis (equal). **Ruisheng Choo:** Data curation (equal); formal analysis (equal). **Li Si Tay:** Data curation (equal); formal analysis (equal). **Sun Yi Soh:** Data curation (equal); formal analysis (equal). **Zaki Jamil:** Data curation (equal). **Wee Foong Ang:** Conceptualization (equal); data curation (equal). **Adrian H. B. Loo:** Conceptualization (equal); data curation (equal).

## CONFLICT OF INTEREST STATEMENT

None declared.

## Data Availability

Data is available at https://doi.org/10.5061/dryad.zs7h44jj0

## APPENDIX A

**TABLE A1:** List of bulbuls that can be found in Singapore, with details on its beak width, gape size, origin, habitat, threat status (IUCN, and 2nd and 3rd edition of the Singapore Red Data Book (the species red list for Singapore)), and occurrence in Singapore.

Common name	Scientific name	Beak width (mm)	Gape size (mm)	Origin	Habitat	Feeding zone within forest	IUCN status	RDB2 status	RDB3 status	Occurrence in Singapore
Straw‐headed Bulbul	*Pycnonotus zeylanicus*	5.1	Not listed	Native	Forest & woodland	Middlestorey	CR	EN	EN	Common resident
Black‐and‐white Bulbul	*Pycnonotus melanoleucos*	4.2	9.8	Native	Forest	Canopy	NT	Not Listed	NT	Very rare non‐breeding visitor
Black‐headed Bulbul	*Pycnonotus atriceps*	4.2	10.2	Native	Forest	Middlestory	LC	CR	CR	Rare resident
Yellow‐vented Bulbul	*Pycnonotus goiavier*	4.3	11.2	Native	Parks & gardens	–	LC	Not Listed	LC	Very common resident
Olive‐winged Bulbul	*Pycnonotus plumosus*	4.7	10	Native	Woodland	Under‐ & middlestorey	LC	Not Listed	LC	Common resident
Cream‐vented Bulbul	*Pycnonotus simplex*	4.0	9.8	Native	Forest	Under‐ & middlestorey	LC	NT	VU	Uncommon resident
Asian Red‐eyed Bulbul	*Pycnonotus brunneus*	4.4	9.9	Native	Forest	Under‐ & middlestorey	LC	EN	VU	Uncommon resident
Buff‐vented Bulbul	*Iole crypta*	5.3	11	Native	Forest	Middlestorey	NT	CR	CR	Very rare non‐breeding visitor
Streaked Bulbul	*Ixos malaccensis*	5.2	Not listed	Native	Forest	Canopy	NT	Not Listed	NT	Rare non‐breeding visitor
Cinereous Bulbul	*Hemixos cinereus*	4.0	Not listed	Native	Forest	Middlestorey & canopy	LC	Not Listed	LC	Uncommon non‐breeding visitor
Black‐crested Bulbul	*Pycnonotus flaviventris*	3.9	8	Introduced	Forest	Middlestorey & canopy	LC	Not Listed	NA	Uncommon introduced resident
Red‐whiskered Bulbul	*Pycnonotus jocosus*	4.5	Not listed	Introduced	Shrubland	–	LC	Not Listed	NA	Uncommon introduced resident
Sooty‐headed Bulbul	*Pycnonotus aurigaster*	4.1	Not listed	Introduced	Shrubland	–	LC	Not Listed	NA	Uncommon introduced resident

*Note*: Feeding zone within forest are from Birds of the World (2022). Origin, habitat, and occurrence data are from Lim et al. (2021). IUCN, RDB2 and RDB3 data are from NParks (2024). Beak width and gape size are from Tobias et al. (2022) and McFadden et al. (2022).

Abbreviations: CR, Critically endangered; EN, Endangered; LC, Least concern; NT, Near threatened.
